# Absence of autoantibodies connected to autoimmune polyendocrine syndrome type I and II and Addison's disease in girls and women with Turner syndrome

**DOI:** 10.1186/1477-5751-6-10

**Published:** 2007-12-18

**Authors:** Annika E Stenberg, Lisskulla Sylvén, Håkan Hedstrand, Olle Kämpe, Malou Hultcrantz

**Affiliations:** 1 Dept. of Otorhinolaryngology, Karolinska University Hospital, Solna, Sweden; 2 Dept. of Woman and Child Health, Karolinska University Hospital, Solna, Sweden; 3 Dept. of Medical Sciences, University Hospital, Uppsala, Sweden

## Abstract

**Background:**

A disturbance in the immune system has been described in Turner syndrome (45,X), with an association to low levels of IgG and IgM and decreased levels of T- and B-lymphocytes. Also different autoimmune diseases have been connected to Turner syndrome (45,X), thyroiditis being the most common. Other autoimmune diseases seen are inflammatory bowel disease, insulin dependent diabetes mellitus, Addison's disease, rheumatoid arthritis, myasthenia gravis, vitiligo, alopecia, pernicious anaemia and hypoparathyroidism, but the association to Turner syndrome is not definite.

Besides the typical features of Turner syndrome (short stature, failure to enter puberty spontaneously and infertility due to ovarian insufficiency) ear problems are common. Otitis media and a progressive sensorineural hearing disorder are commonly seen. In the normal population there are known inner ear disorders related to autoimmune diseases. The aim of this study was to investigate patients with Turner syndrome regarding autoantibodies connected to the autoimmune disorders; autoimmune polyendocrine syndrome type I and II and Addison's disease, to screen for overlapping profile of autoantibodies.

Blood samples from 110 Turner patients (7–65 years) were investigated using *in vitro *transcription, translation and immunoprecipitation techniques regarding autoantibodies connected to autoimmune polyendocrine syndrome type I and II and Addison's disease (21-hydroxylase, 17α-hydroxylase, side-chain cleavage enzyme, aromatic L-amino acid decarboxylase, tyrosine hydroxylase and tryptophan hydroxylase).

**Results:**

The autoantibodies investigated were not overrepresented among the Turner patients.

**Conclusion:**

The autoimmune disorders associated with Turner syndrome do not seem to be of the same origin as Addison's disease, the type I or II autoimmune polyendocrine syndrome.

## Background

Turner syndrome is caused by the presence of only one normally functioning X-chromosome. The other sex chromosome can be missing (45,X) or abnormal and mosaicism is often present. Occurring in one of every 2000 female births, Turner syndrome is one of our most common sex chromosome abnormalities [1]. Turner syndrome is characterized by short stature, no spontaneous puberty and infertility due to ovarian dysgenesis [2]. Besides the typical features of Turner syndrome ear problems are common. During childhood the girls repeatedly suffer from otitis media and a progressive sensorineural hearing disorder is commonly seen [3-6].

Immunological disturbances have previously been described in Turner syndrome, with an association to reduced levels of serum IgG and IgM, increased IgA and decreased levels of circulating T- and B-lymphocytes. However the results have not been conclusive [6-9].

Also different autoimmune diseases have been connected to Turner syndrome. An increased incidence of anti-thyroid antibodies has repeatedly been reported and thyroid dysfunctions are common [2,10-12]. Other autoimmune diseases described are inflammatory bowel disease, insulin dependent diabetes mellitus (IDDM), Addison's disease, rheumatoid arthritis, myasthenia gravis, vitiligo, alopecia, pernicious anaemia and hypoparathyroidism, but the association to Turner syndrome is not definite [10].

Normally Addison's disease is often associated with other autoimmune diseases, such as thyroiditis, IDDM, premature ovarian failure (POF), vitiligo and hypoparathyroidism. Addison's disease may be present as an isolated disorder or part of type I or II autoimmune polyendocrine syndrome (APS I or APS II), where also pernicious anaemia, gastrointestinal dysfunctions, alopecia and chronic candidiasis are common [13]. Several autoantigens have been identified to be connected to these three disorders as illustrated in table 1[13-18]. Autoimmune inner ear disease is a multifactorial disorder that lately has been discussed. These patients often present autoantibodies or cellular factors, directed against several inner ear structures, but the etiology is still not completely characterized [19-21]. Of the autoantibodies seen in APS I, tyrosine hydroxylase (TH) could be a possible cochlear autoantigen, as it has been proposed as potential melanin catalyser in the inner ear [22]. Some inner ear diseases are known to be caused by water and ion regulating problems, which is the case with Addison's disease.

**Table 1:** 

**Disease**	**Antigen**	**Enzyme action**
Addison's disease	21-Hydroxylase (21-OH)	Steroid hormone synthesis

Addison's disease with POF	21-Hydroxylase (21-OH)	Steroid hormone synthesis
	Side-chain cleavage enzyme (SCC)	Steroid hormone synthesis

APS I	17α-hydroxylase (17α-OH)	Steroid hormone synthesis
	Aromatic L-amino acid decarboxylase (AADC)	Monoaminergic and serotonergic biosynthetic pathways
	Tyrosine hydroxylase (TH)	Rate-limiting enzyme in catecholamine biosynthesis
	Tryptophan hydroxylase (TPH)	Rate limiting enzyme in the synthesis of serotonin

APS II	21-Hydroxylase (21-OH)	Steroid hormone synthesis

Description of important autoantigens in Addison's disease, Addison's disease with premature ovarian failure (POF), APS I and APS II. Patients with Addison's disease generally display autoantibodies to the enzyme 21-OH restricted to the adrenal cortex, while most of the patients with APS I show autoantibodies to SCC, located both in the adrenal cortex and steroid producing cells in the gonads, reflecting the risk of developing ovarian failure. In addition patients with Addison's disease *and *POF, also show antibodies directed against SCC (13). TPH is found as an intestinal autoantigen in APS I patients with intestinal dysfunction (14) and TH autoantibodies are correlated to alopecia areata in these patients (15).

The aim of this study was to investigate girls and women with Turner syndrome regarding autoantibodies connected to the autoimmune disorders APS 1, APS II and Addison's disease, to screen for overlapping profile of autoantibodies.

## Results

### Autoantibodies in Turner women

No autoantibodies to 21-hydroxylase (21-OH), 17α-hydroxylase (17-OH), side-chain cleavage enzyme (SCC), aromatic L-amino acid decarboxylase (AADC) were detected among the Turner women (N = 110). However, one Turner woman presented autoantibodies to tryptophan hydroxylase (TPH) and two to tyrosine hydroxylase (TH).

Except for 3 blood donors having low titers of antibodies to TH, none of the 40 blood donors showed any positive autoantibody titers.

Antibodies to thyroid peroxidase (TPO) were recorded in 40% (36/91) of the Turner women analyzed, and of these 50% had hypothyroidism. Of the women who were positive to anti-TPO, only 6 showed a hearing loss, as compared 16 women in the group negative to anti-TPO.

## Discussion

When screening for autoantibodies the only autoantibody overrepresented was that against TPO (40%), correlating to an increased incidence of thyroid dysfunctions among the Turner patients. This is in concordance with previously described prevalence of thyroiditis (20–50%). However in earlier studies even higher prevalence of anti-thyroid autoantibodies (50–85%) has been observed [10]. This discrepancy could depend on differences in patient selection and/or methods. There is a known connection between hypothyroidism and hearing dysfunction [23]. However, hearing loss was not over represented in the group with anti-TPO antibodies.

Considering that many of the autoimmune diseases documented in Turner syndrome [10-12] also are seen in APS I and APS II, one could imagine an overlapping autoantibody profile. In this study the only autoantibodies found in Turner syndrome were directed against TH and TPH, in two respectively one woman with Turner syndrome. TH autoantibodies were also found in a few healthy blood donors. Antibodies to 21-OH, the most important autoantigen in Addison's disease [13], were not registered in any of the women with Turner syndrome, which is in line with that none of the examined patients presented any symptoms of Addison's disease. Only a few other autoimmune diseases were present in the group of Turner patient (see materials and methods), however these data were obtained through the medical history. Consequently a putative relation between Turner syndrome and the autoimmunity seen in APS I or APS II seems weak.

Turner females have a progressive hearing loss, both in the midfrequencies and also in the high frequencies [3,4], why an autoimmune situation could be suspected. In this study the only potential cochlear antigen investigated was TH. As TH autoantibodies were found only in two patients, not correlating to a hearing disability, TH does not appear to be an autoantigen connected to the sensorineural hearing loss seen in Turner syndrome. One hypothesis, put forward by Barrenäs et al., is that the ear and hearing problems are correlated to the degree of X chromosome loss, leading to growth disturbances during fetal life [24]. An additional theory discussed is that the age dependent sensorineural hearing impairment may be enhanced because of the lack of estrogens [25].

## Conclusion

In conclusion the autoimmune disorders sometimes associated with Turner syndrome do not seem to be of the same origin as Addison's disease, APS I or APS II.

## Methods

### Subjects

Blood samples from patients with the diagnosis Turner syndrome, genetically confirmed, were investigated according to the Swedish ethical record no 88–265.

The patients consisted of 110 girls and women with Turner syndrome in the Stockholm area aged 7–65 years (median age 33 years). Autoimmune diseases present in women older than 18 years (n = 97) were thyroid dysfunction (23%), celiac disease (4%), inflammatory bowel disease (3%), diabetes melittus (IDDM) (3%), and Vitiligo (1%). The karyotypes of the patients were: 45,X (51%), 45,X/46,XX (23%), 45,X/46,XY (3%), 45,X/46,XX/47,XXX (3%), 45,X/46,X,i(Xq) (18%) and 45,X/46,X,r(X) (3%) (r = ring chromosome). In the group 45,X/46,XX the karyotypes 45,X/46,X,del(X)(q11) (del = deletion) and 45,X/46,XXq+ were included. Forty healthy sex and age matched blood donors served as controls.

A medical history was attained, focusing on autoimmune diseases, previous and current ear diseases, ear operations and hearing problems. A clinical investigation of the Ear-Nose and Throat area was also performed.

### In vitro transcription and translation and immunoprecipitation

Autoantibodies to 21-hydroxylase (21-OH), 17α-hydroxylase (17-OH), side-chain cleavage enzyme (SCC), aromatic L-amino acid decarboxylase (AADC), tyrosine hydroxylase (TH) and tryptophan hydroxylase (TPH) were analysed as follows:

#### In vitro transcription and translation and immunoprecipitation

Plasmids, containing cDNA of the antigens were purified with Qiagen miniprep kit (Qiagen GmbH, Hilden, Germany). The construction of the plasmids has been published elsewhere [14-18]. *In vitro *transcription and translation of the purified plasmids were performed using the TNT SP6 and T3-coupled reticulocyte lysate system (Promega). The correct size of the radioactive product was analyzed on a SDS-PAGE minigel (BioRad, Richmond, CA) according to standard protocols. Each [^35^S]-radiolabeled protein (21-OH, 17-OH, SCC, AADC, TH and TPH) was used for immunoprecipitation with patient sera in a 96-wells plate assay as described elsewhere [14-18]. The results were expressed as index ((sample - cpm negative control)/(cpm positive control - cpm negative control) × 100). Each sample was analyzed in duplicates. Sera from APS I, II and Addison patients known to have antibodies against each antigen were used as positive controls and one of the blood donors was used as a negative control in each microwell plate. The upper normal limit of each antibody index, which was the mean value for 28 blood donors plus 3 standard deviations, was calculated. Sera from 40 additional blood donors were also used as blind negative controls.

In addition 91 of the Turner patient sera were screened for autoantibodies against thyroxin peroxidase (TPO) using routine methods at the Div. of Clinical immunology, Karolinska Hospital, Stockholm, Sweden.

## Competing interests

The authors declare that they have no competing interests.

## Authors' contributions

AES participated in the design of the study, analyzed the results and drafted the manuscript. LS participated in the design of the study and collected the blood samples. HH and OK performed the *In vitro *transcription and translation and immunoprecipitation and analyzed the results. MH participated in the design and coordination of the study and collected the blood samples.

All authors read and approved the final manuscript.
